# Influence of the Different Primary Cancers and Different Types of Bone Metastasis on the Lesion-based Artificial Neural Network Value Calculated by a Computer-aided Diagnostic System, BONENAVI, on Bone Scintigraphy Images

**DOI:** 10.22038/aojnmb.2016.7606

**Published:** 2017

**Authors:** Takuro Isoda, Shingo BaBa, Yasuhiro Maruoka, Yoshiyuki Kitamura, Keiichiro Tahara, Masayuki Sasaki, Masamitsu Hatakenaka, Hiroshi Honda

**Affiliations:** 1 Department of Clinical Radiology, Graduate School of Medical Sciences, Kyushu University, Fukuoka, Japan; 2 Department of Health Sciences, Graduate School of Medical Sciences, Kyushu University, Fukuoka, Japan; 3 Department of Diagnostic Radiology, Sapporo Medical University, Hokkaido, Japan

**Keywords:** Bone metastasis, Bone scintigraphy, BONENAVI Computer-aided diagnosis

## Abstract

**Objective(s):**

BONENAVI, a computer-aided diagnostic system, is used in bone scintigraphy. This system provides the artificial neural network (ANN) and bone scan index (BSI) values. ANN is associated with the possibility of bone metastasis, while BSI is related to the amount of bone metastasis. The degree of uptake on bone scintigraphy can be affected by the type of bone metastasis. Therefore, the ANN value provided by BONENAVI may be influenced by the characteristics of bone metastasis. In this study, we aimed to assess the relationship between ANN value and characteristics of bone metastasis.

**Methods:**

We analyzed 50 patients (36 males,14 females; age range: 87–42 yrs median age:72.5 yrs) with prostate, breast, or lung cancer who had undergone bone scintigraphy and were diagnosed with bone metastasis (32 cases of prostate cancer, nine cases of breast cancer, and nine cases of lung cancer). Those who had received systematic therapy over the past years were excluded. Bone metastases were diagnosed clinically, and the type of bone metastasis (osteoblastic, mildly osteoblastic, osteolytic, and mixed components) was decided visually by the agreement of two radiologists. We compared the ANN values (case-based and lesion-based) among the three primary cancers and four types of bone metastasis.

**Results:**

There was no significant difference in case-based ANN values among prostate, breast, and lung cancers. However, the lesion-based ANN values were the highest in cases with prostate cancer and the lowest in cases of lung cancer (median values: prostate cancer, 0.980; breast cancer 0.909; and lung cancer, 0.864). Mildly osteoblastic lesions showed significantly lower ANN values than the other three types of bone metastasis (median values: osteoblastic,; 0.939 mildly osteoblastic; 0.788, mixed type; 0.991, and osteolytic. 0.969) The possibility of a lesion-based ANN value below 0.5 was %10.9 for bone metastasis in prostate cancer, %12.9 for breast cancer, and %37.2 for lung cancer. The corresponding possibility were %14.7 for osteoblastic metastases, %23.9 for mildly osteoblastic metastases, %7.14 for mixed-type metastases, and %16.0 for osteolytic metastases.

**Conclusion:**

The lesion-based ANN values calculated by BONENAVI can be influenced by the type of primary cancer and bone metastasis.

## Introduction

Bone scintigraphy is used in tumor staging to detect bone metastasis and to obtain information about the extent of bone metastasis. The extent of disease has been long used as a semi-quantitative grading system to evaluate the extent of skeletal metastasis in patients with prostate cancer (1).

The bone scan index (BSI) is a quantitative parameter proposed to analyze the extent of bone metastasis in patients with prostate cancer (2). BSI shows the extent of hot spots as the percentage of the entire bone and can be used to follow-up patients with bone metastasis due to prostate cancer. Overall, BSI in patients with prostate cancer and osseous lesions is a prognostic marker (3-5).

The computer-aided diagnostic system, EXINIbone (EXINI Diagnostics AB, Lund, Sweden), was developed for use in bone scintigraphy (6, 7). BONENAVI (Fujifilm RI Pharma Co., Ltd., Tokyo, Japan) uses the same system as EXINIbone, along with a Japanese database for supervised learning (8-13). BONENAVI provides the following two parameters for case-based analyses: artificial neural network (ANN) and BSI; BONENAVI also calculates the ANN and BSI for each lesion.

ANN indicates the possibility of bone metastasis as a value ranging between 0 and 1. Cases with an ANN value ≥0.5 are estimated to have bone metastasis, and hot spots showing an ANN value ≥0.5 are speculated to indicate a metastasized lesion. On the other hand, the BSI of each hot spot indicates the ratio (%) of the hot spot area to the entire bone.

BSI for a case is the sum of BSI values of hot spots with high ANN values (≥0.5). It is important to calculate the correct lesion-based ANN, as BSI for each case is the sum of BSIs of hot spots with high lesion-based ANN values (≥0.5). Also, the case-based BSI could change when the lesion-based ANN is either underestimated or overestimated.

The degree of uptake on bone scintigraphy generally depends on the type of bone metastasis, such as osteolytic or osteoblastic bone metastasis. An osteolytic lesion tends to show a lower uptake, compared to an osteoblastic lesion. According to a recent report, acquisition time affects the calculation of BSI with BONENAVI (14). In this study, we hypothesized that the type of bone metastasis might also influence the ANN value of lesions calculated by BONENAVI. We also analyzed the relationship between ANN values and type of bone metastasis.

## Methods

### Patients

This retrospective study was approved by the review board of our institution, and the requirement of written informed consent was waived. We enrolled a total of 50 patients with bone metastasis (32 patients with prostate cancer, nine patients with breast cancer, and nine patients with lung cancer; male: female ratio= 36:14), who underwent bone scintigraphy and were diagnosed with bone metastasis between October 2007 and June 2014. The patients’ age ranged from 42 to 87 years (median: 72.5 yrs).

Bone metastasis was diagnosed clinically, using the following modalities: X-ray computed tomography (CT), magnetic resonance imaging, and F-18 fluorodeoxyglucose positron emission tomography (FDG-PET)/CT scan.

### Bone scintigraphy

Bone scintigraphy was performed 4 h after the administration of 740 MBq of ^99m^Tc methylene diphosphonate (MDP, Fujifilm RI Pharma Co., Ltd., Tokyo, Japan). Whole-body scintigraphy was performed, using a dual-head gamma camera (Symbia T6, Siemens Healthcare, Malvern, PA, USA). Anterior and posterior images were acquired at a speed of 15 cm/min with low-energy high-resolution (LEHR) collimators, a 256×1024 pixel size, and a 140-keV photopeak with a 20% window. SPECT/CT was used to find the bone lesions on CT which matched hot spots on bone scintigraphy.

### SPECT/CT protocol

Single-photon emission computed tomography (SPECT)/CT images were obtained, using a gamma camera with six-section spiral CT images within the same gantry. SPECT/CT scans for regions with abnormal uptake on planar images were obtained. SPECT images were acquired in the step-and-shoot mode with 72 projections (duration of 10 sec for each projection), a non-circular orbit over 360°, LEHR collimators, a 128×128 matrix, and a 140-keV photopeak with a 20% window.

Three-dimensional ordered subset expectation maximization iterative reconstructions was applied using four iterations and eight subsets. CT-based attenuation correction without scatter correction was applied to the SPECT images. The CT scan parameters were 130 keV, 30 mAs or less (due to the minimization of radiation exposure), a 512×512 matrix, 2×2.5 mm collimation, and 5-mm section thickness.

### BONENAVI

BONENAVI version 2.1.6 was used in this study. The database consisted of 1532 patients from nine Japanese hospitals. In total, 42% of the patients had bone metastasis, including cases with prostate cancer (29%), cases with breast cancer (41%), and others (30%) (9).

**Figure 1:**
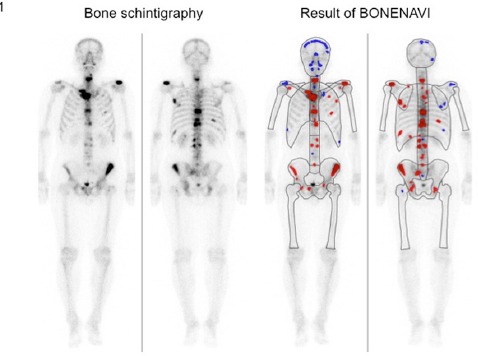
Bone scintigraphy of a patient with bone metastasis from prostate cancer and the results of analysis by BONENAVI. The spots shown in red are those with high ANN values, (≥ 0.5) and the spots in blue are those with low ANN values (<0.5)

BONENAVI automatically performed segme-ntation (12 regions) and detected hotspots on the anterior and posterior images (Figure 1). Overall, BONENAVI calculates the values of ANN (case-based and lesion-based) and BSI (case-based and lesion-based). As noted earlier, ANN is a parameter indicating the possibility of bone metastasis, and ranges between 0 and 1. This parameter was calculated for each hot spot and each case. A lesion or a case was considered as bone metastasis when its ANN was ≥0.5.

BONENAVI uses 45 features including the size, intensity, shape, and location of hot spots to calculate lesion-based ANN and uses 26 features to calculate case-based ANN. On bone scintigraphy, hot spots are shown in blue (ANN<0.5) or red (ANN≥0.5), depending on the ANN value (Figure 1).

### Analysis

All lesions, detected individually on bone scintigraphy and confirmed by other imaging inspections, were included in the analysis. Metastatic bone lesions were excluded if their uptake on bone scintigraphy was fused with others. We compared the ANN values for a case and a lesion among three types of primary cancer, i.e., prostate, breast, and lung cancers.

**Figure 2:**
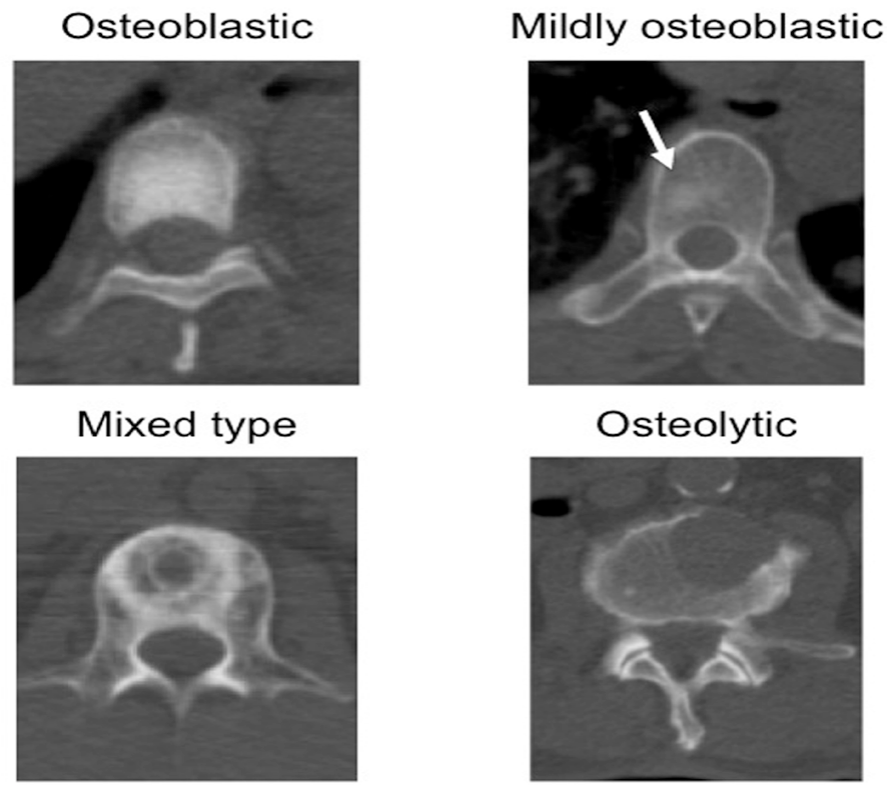
Typical examples of four types of bone metastasis. The arrow indicates a mildly osteoblastic area in the vertebral body

Metastatic bone lesions were visually classified into four types by the agreement of two radiologists: osteoblastic, mildly osteoblastic, mixed osteoblastic/osteolytic, and osteolytic lesions (Table 1, Figure 2). Osteoblastic and osteolytic lesions should not share more than 30% of the area with other components. Mildly osteoblastic lesions show a slightly higher density, compared to the normal bone and include <30% osteolytic area. On the other hand, mixed lesions contain both >30% osteoblastic area and >30% osteolytic area.

**Table 1:** The difference in lesion-based artificial neural network (ANN), lesion-based bone scan index (BSI), and CT values among primary cancers and different types of bone metastasis

	Osteoblastic	Mildly osteoblastic	Mixed type	Osteolytic	Total
Prostate					

ANN	0.950	0.802	0.992	0.998	0.980
BSI	0.251	0.089	0.184	0.174	0.198
CT value (HU)	500.4	355.1	267.0	145.7*	358.2**
Number	79	25	33	37	174

Breast					

ANN	0.925	0.779	0.981	0.910	0.909
BSI	0.333	0.108	0.427	0.161	0.229
CT value (HU)	644.3	277.7	322.8	133.4	320.5**
Number	21	18	6	17	62

Lung					

ANN	0.922	0.593	0.928	0.734	0.864
BSI	0.120	0.142	0.132	0.133	0.129
CT value (HU)	520.5	123.1	391.2	98.5*	193.2**
Number	16	3	3	21	43

Total					

ANN	0.939	0.788	0.991	0.969	0.945
BSI	0.119***	0.076***	0.111***	0.083	0.103
CT value (HU)	523.2	320.5	269.7	131.7	347.8
Number	116	46	42	75	279

The median values of lesion-based ANN, lesion-based BSI, and CT are indicated. The number of analyzed lesions is also shown

* The CT value of osteolytic bone metastasis was significantly higher in prostate cancer than lung cancer (P=0.0159)

** The CT value of bone metastasis was significantly lower in lung cancer than prostate and breast cancers (P=0.0049 and 0.0395, respectively)

*** The BSI of mildly osteoblastic lesions was significantly lower than osteoblastic and mixed-type metastases (P=0.0020 and 0.0031, respectively)

lowest ANN values among the four types of bone metastasis with significant differences

Hot spots with normal CT findings (neither the osteoblastic nor the osteolytic area occupied 30% or more of the hot spot region) were excluded from the present analysis. The CT value (mean) of the lesions was measured on a medical imaging and information management system, i.e., SYNAPSE (FUJIFILM Medical Co., Ltd., Tokyo, Japan). The lesion-based ANN values were compared among the four types of bone metastasis. The ANN value of hot spots, which BONENAVI could not detect, was set at 0.

### Statistical analysis

Statistical analyses were performed, using JMP 11 software (SAS Institute, Cary, SC, USA). Wilcoxon signed-rank test and Chi-square test were used to analyze the difference among different types of primary cancer or bone metastasis. P-value less than 0.05 was considered statistically significant.

## Results

**Figure 3:**
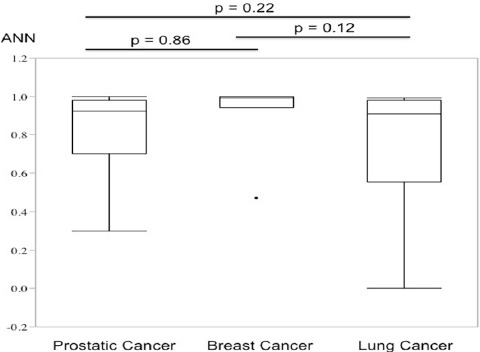
Comparison of case-based ANN values among prostate, breast, and lung cancers. The upper extreme, upper quartile, median, lower quartile, lower extreme, and outlier are indicated in the boxplot. Breast cancer showed the highest ANN values when comparing the medians, although there was no significant difference

In the case-based analysis, the ANN values among prostate, breast, and lung cancers were compared. The median ANN values were 0.925, 0.990, and 0.911 for prostate, breast, and lung cancers, respectively, with no significant difference among these primary cancers (Figure 3). Five out of 50 cases showed a case-based ANN value <0.5 and were misdiagnosed as showing a low possibility of bone metastasis. Among these five cases, two were prostate cancer (two out of 32 cases with prostate cancer, 6.25%), two were lung cancer (two out of nine cases, 22.2%), and one was breast cancer (one out of nine cases, 11.1%).

**Figure 4:**
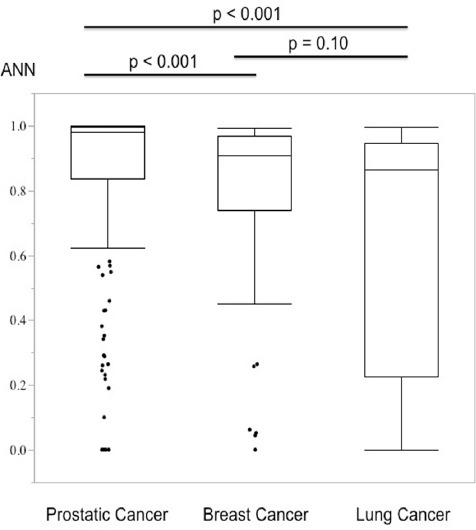
Comparison of lesion-based ANN values among prostate, breast, and lung cancers. Prostate cancer showed the highest ANN values with a significant difference

In the analysis of the lesions, 174 lesions in prostate cancer, 62 lesions in breast cancer, and 43 lesions in lung cancer were analyzed (Table 1). The ANN values of prostate cancer were significantly higher than the other two primary cancers (median values: prostate cancer, 0.980; breast cancer, 0.909; and lung cancer, 0.864) (Table 1, Figure 4).

**Figure 5:**
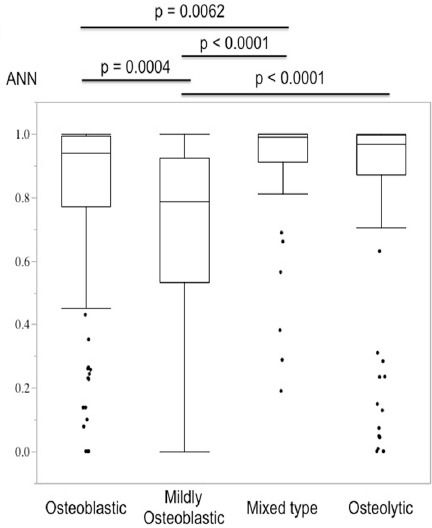
Comparison of lesion-based ANN values among osteoblastic, mildly osteoblastic, mixed-type, and osteolytic lesions. Mildly osteoblastic lesions showed the lowest ANN values among the four types of bone metastasis with significant differences

There was no significant difference in lesion-based ANN values between breast and lung cancers. Lesions with an ANN value <0.5 is considered to have low possibility of bone metastasis and were excluded from the calculation of case-based BSI. The possibility of a lesion-based ANN value <0.5 was 10.9%, 12.9%, and 37.2% for bone metastases due to prostate, breast, and lung cancers, respectively.

Bone metastasis from lung cancer showed the highest possibility of the underestimation of lesion-based ANN, with a significant difference between the three primary cancers (P=0.0005, Chi-square test). The lesion-based BSI and CT values of metastatic bone lesions were also analyzed. There was no significant difference in lesion-based BSI among prostate (0.198), breast (0.229), and lung (0.103) cancers (Table 1). The CT value of bone metastasis was lower in lung cancer (193.2 HU) in comparison with prostate (358.2 HU, P=0.0049) and breast (320.5 HU, P=0.0395) cancers.

We then classified metastatic bone lesions into four groups (osteoblastic, mildly osteoblastic, mixed, and osteolytic). Mildly osteoblastic lesions (median: 0.788) showed the lowest lesion-based ANN values, while the mixed-type lesions (median: 0.991) showed the highest lesion-based ANN values among the four types of bone metastasis (Table 1, Figure 4).

The ANN values of mildly osteoblastic lesions were significantly lower than those of osteoblastic (median: 0.939), mixed-type (median: 0.991), and osteolytic (median: 0.969) lesions. There was also a significant difference between the osteoblastic and mixed-type lesions (Table 1, Figure 4). Mildly osteoblastic lesions showed the lowest ANN values in all three types of primary cancers.

In contrast, osteolytic lesions showed inconsistent results. They showed the highest ANN values in prostate cancer and the second lowest values in lung cancer (Table 1). The lesion-based BSI of mildly osteoblastic lesions was lower than the values in osteoblastic (P=0.0020) and mixed-type (P=0.0031) lesions. There was no significant difference in other combinations of the four types of bone metastasis.

Osteoblastic lesions showed the highest CT value (523.2 HU), while osteolytic lesions showed the lowest CT value (131.7 HU) among the four types of bone metastasis. In osteolytic lesions, bone metastasis from prostate cancer (145.7 HU) showed a significantly higher CT value, compared to lung cancer (98.5 HU, P=0.0159). Bone metastasis from prostate cancer showed the highest ANN values in all four types of bone metastasis (osteoblastic, mildly osteoblastic, mixed-type, and osteolytic bone metastases) among the three primary cancers (Table 1).

The possibility of a lesion-based ANN value <0.5 was 14.7% for osteoblastic, 23.9% for mildly osteoblastic, 7.1% for mixed-type, and 16.0% for osteolytic bone metastases. Mildly osteoblastic lesions showed the highest possibility of misdiagnosis, although there was no significant difference among the four types of bone metastasis (Chi-square test, P=0.185).

## Discussion

BSI is a quantitative marker for the spread of bone metastasis and is widely used as a prognostic and response indicator (4). The calculation of case-based BSI by BONENAVI is performed by summing the lesion-based BSI values with high ANN values (≥ 0.5). It is very important to calculate the lesion-based ANN correctly for the accuracy of case-based BSI, since the lesion is excluded from the calculation of case-based BSI if the lesion-based ANN value is underestimated (resulting in a value lower than 0.5).

In the present study, there was no significant difference in the accuracy of diagnosing bone metastasis among prostate, breast, and lung cancers when the case-based analysis was performed. However, metastatic bone lesions from prostate cancer patients showed significantly higher lesion-based ANN values, compared to breast and lung cancer patients.

Moreover, prostate cancer showed the highest ANN values of all four types of bone metastasis (osteoblastic, mildly osteoblastic, mixed-type, and osteolytic bone metastases) among the three primary cancers. This result indicated that some factors are involved in the elevation of ANN value in bone metastasis from prostate cancer, regardless of the type of bone metastasis.

A lesion-based BSI is related to the size of bone metastasis (also uptake intensity), and a high CT value generally results in high uptake intensity on bone scintigraphy. We compared lesion-based BSI and CT values among three types of primary cancers. However, the reason why bone metastasis from prostate cancer showed high ANN value could not be explained in this study. Other features on bone scintigraphy such as pixel value (maximum value and SD), symmetry, and skeletal region probably affected the high lesion-based ANN value of bone metastasis in prostate cancer (6).

Bone metastases from lung cancer showed the lowest lesion-based ANN values among the three primary cancers, and there was no significant difference between breast and lung cancers. In addition, metastatic bone lesions from lung cancer showed <0.5 lesion-based ANN values more frequently than prostate and breast cancers. Case-based BSI is calculated by summing the lesion-based BSI with high ANN values (≥0.5). This finding indicates that the case-based BSI of lung cancer would be underestimated more frequently, compared to prostate and breast cancers.

One of the reasons why lesion-based ANN values in lung cancer cases were lower than those of the other two types of primary cancers might be the low CT value of bone metastasis (Table 1). Also, the supervised learning in the BONENAVI system might not be adequate for lung cancer, compared to prostate and breast cancers. This is because BONENAVI uses a Japanese database in which prostate and breast cancers account for 70% of the total cases, while lung cancer is only one of the other primary cancers in the remaining 30% of the database (9).

Mildly osteoblastic lesions showed the lowest ANN values in all three types of primary cancers. This result implies that the mildly osteoblastic type itself is a factor for low ANN values. Mildly osteoblastic lesions tended to show a low uptake and exhibited small lesion-based BSI values in this study (Table 1), which is probably one of the reasons why mildly osteoblastic lesions showed the lowest ANN values.

On the other hand, osteolytic lesions in prostate cancer showed high ANN values, whereas those in lung cancer showed low ANN values. In this study, osteolytic lesions were defined as having an osteoblastic component which was not >30%; in other words, an osteolytic lesion could contain a small amount of osteoblastic area.

In the present analysis, osteolytic lesions in prostate cancer were disposed to have more osteoblastic components, compared to those in lung cancer, although the amount of osteoblastic component was not high enough to be classified as a mixed-type lesion.

In the comparison of CT values, osteolytic bone metastasis of prostate cancer showed a significantly higher CT value than that of lung cancer (Table 1). This difference in the amount of osteoblastic component between prostate and lung cancers probably resulted in the difference in lesion-based ANN values.

Our results also revealed that lung cancer cases showed lesion-based ANN values < 0.5 more frequently than prostate and breast cancers. One of the reasons for this underestimation could be the lower CT value of bone metastasis in lung cancer than prostate and breast cancers. In other words, bone metastasis from lung cancer had less osteoblastic components, compared to the other two cancers.

One of the limitations of the present study was the retrospective design, although consecutive patients who underwent bone scintigraphy for cancer staging were included. The relatively small number of patients and the clinical diagnosis of bone metastasis are also other limitations of this study. However, histopathological confirmation of bone metastasis is generally impractical due to the advanced stage of patients, unless it has a strong clinical impact.

## Conclusion

Lesion-based ANN values calculated by BONENAVI might be influenced by the type of primary cancer and bone metastasis. Mildly osteoblastic lesions and bone metastasis from lung cancer tended to show lower lesion-based ANN values, compared to other types of bone metastasis and primary cancers, thereby leading to the underestimation of case-based BSI values.
